# RNA-binding protein family diversification correlates with neural complexity across metazoan evolution

**DOI:** 10.1016/j.isci.2026.115766

**Published:** 2026-04-17

**Authors:** Kyota Yasuda

**Affiliations:** 1 Graduate School of Integrated Sciences for Life, Hiroshima University, 1-3-1 Kagamiyama, Higashi-Hiroshima, Hiroshima 739-8526, Japan; 2 International Institute for Sustainability with Knotted Chiral Meta Matter (SKCM2), Hiroshima University, 1-3-1 Kagamiyama, Higashi-Hiroshima, Hiroshima 739-8526, Japan; 3 Research Center for the Mathematics on Chromatin Live Dynamics (RcMcD), Hiroshima University, 1-3-1 Kagamiyama, Higashi-Hiroshima, Hiroshima 739-8531, Japan

**Keywords:** Evolutionary biology, Systems biology

## Abstract

RNA-binding proteins (RBPs) control post-transcriptional gene expression with critical roles in nervous system function. Here, we ask whether RBP family diversity tracks with neural complexity across animal evolution. Comparing six species ranging from a simple worm to humans, we find that the number of distinct RBP domain families increases progressively with neuron number—a relationship specific to RBPs and not seen for kinases or G-protein-coupled receptors. Transcription factors show a different pattern, reaching a ceiling in vertebrates while RBP families continue expanding. We further show that the disordered structural regions of RBPs grow longer in more complex organisms, whereas liquid-liquid phase separation propensity remains broadly conserved. Finally, the lengthening of messenger RNA regulatory tails parallels RBP diversification, suggesting co-evolution of post-transcriptional regulatory capacity. These findings establish RBP family diversification as a distinctive molecular signature of animal neural complexity.

## Highlights

• RBP family diversity correlates with neural complexity (ρ = 0.886, p = 0.019)
• RBP-specific; transcription factors saturate at 72 families across vertebrates
• IDR length expands with complexity (46→78 aa; ρ = 0.943); LLPS propensity conserved
• 3′UTR length co-evolves with RBP diversity; 5′UTR and CDS lengths do not

## Graphical abstract

**Figure:**
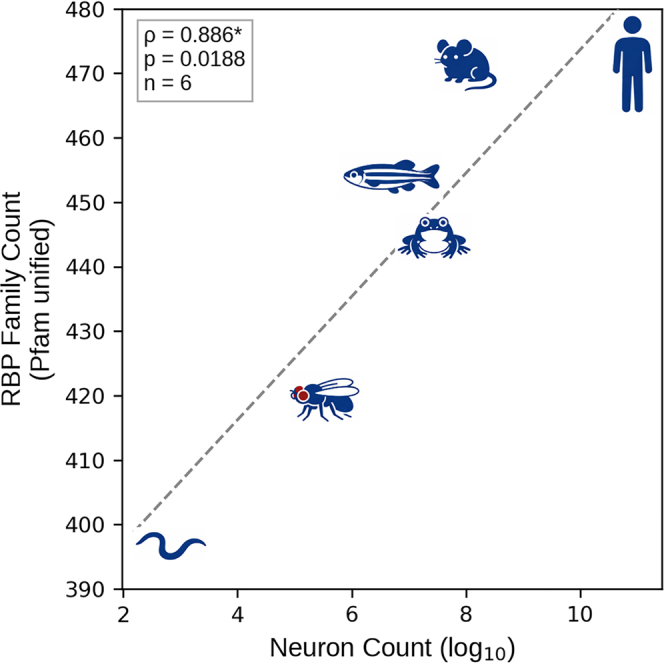

## Introduction

The evolution of biological complexity remains a central question in evolutionary biology. While increases in organismal complexity have been associated with the expansion of gene regulatory networks, the relative contributions of transcriptional versus post-transcriptional regulatory mechanisms remain incompletely understood. The nervous system presents a particularly compelling context for examining this question, as neurons exhibit exceptional requirements for spatial and temporal control of gene expression to support their elaborate morphology, extensive connectivity, and capacity for synaptic plasticity.^1^

RNA-binding proteins (RBPs) constitute a critical layer of post-transcriptional gene regulation, controlling mRNA processing, localization, stability, and translation.^2^^,^^3^^,^^4^ In neurons, RBPs enable precise regulation of protein synthesis at synapses, subcellular targeting of specific transcripts to dendrites and axons, and dynamic responses to synaptic activity—functions essential for neural development, synaptic transmission, and cognitive processes.^5^^,^^6^^,^^7^ Disruption of RBP function causes a spectrum of neurological diseases, including fragile X syndrome, spinal muscular atrophy, and amyotrophic lateral sclerosis,^8^^,^^9^^,^^10^^,^^11^^,^^12^ underscoring their essential role in nervous system function.

The species analyzed in this study span more than six orders of magnitude in neuron number (302 in *C. elegans* to approximately 86 billion in *H. sapiens*), providing substantial dynamic range for correlative analysis. Vertebrates possess substantially more RBP-encoding genes than invertebrates, an expansion linked to whole-genome duplications (WGDs) early in vertebrate evolution.^13^^,^^14^^,^^15^ While consequences of WGD for transcription factor families have been extensively studied, the specific impact on RBP family diversity and its relationship to organismal complexity has not been systematically quantified.^13^^,^^14^^,^^15^^,^^16^

Recent work has highlighted liquid-liquid phase separation (LLPS) as a biophysical mechanism underlying RBP function, particularly in the formation of membraneless organelles such as stress granules and neuronal transport granules.^17^^,^^18^^,^^19^ However, whether LLPS capacity drives RBP family expansion during evolution, or represents one conserved functional mechanism among many, remains unexplored.

To address these questions, we performed a systematic comparative analysis of RBP family diversity across six metazoan species: *Caenorhabditis elegans*, *Drosophila melanogaster*, *Danio rerio*, *Xenopus tropicalis*, *Mus musculus*, and *Homo sapiens*. We used a unified Pfam domain-based classification anchored to EuRBPDB, analyzed LLPS propensity, intrinsically disordered regions, 3′UTR length co-evolution, and non-canonical RBPs, and compared RBP patterns to control protein classes.

## Results

### RBP family diversity strongly correlates with organismal complexity

**Figure 1:**
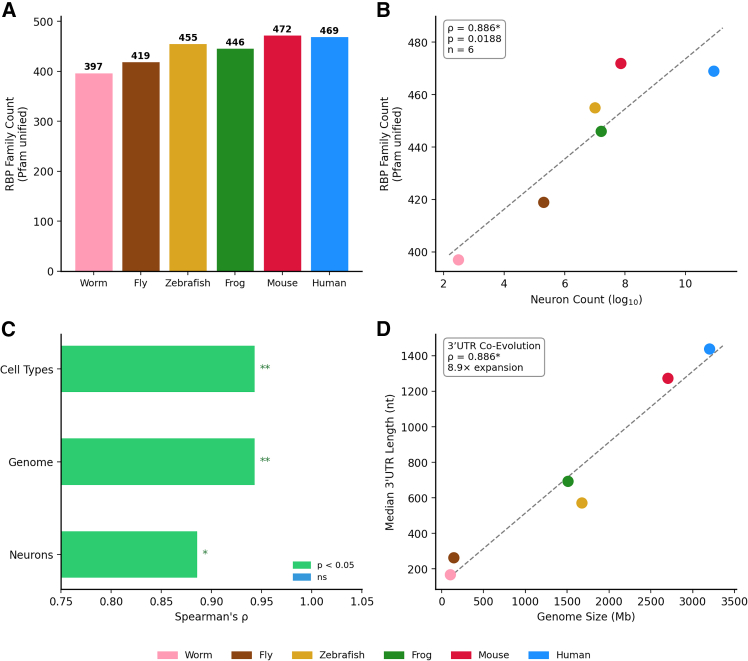
RBP Pfam family diversity correlates with neural complexity (A) RBP Pfam domain family diversity across six species (worm: 397, fly: 419, zebrafish: 455, frog: 446, mouse: 472, human: 469). (B) Spearman’s correlation of RBP family diversity vs. neuronal count (ρ = 0.886, *p* = 0.019). Neuronal counts: *C. elegans*, White et al. (1986); *D. melanogaster*, Raji & Potter (2021); *D. rerio*, Hinsch & Zupanc (2007); *X. tropicalis*, Kemali & Braitenberg (1969); *M. musculus*, Herculano-Houzel et al. (2007); *H. sapiens*, Azevedo et al. (2009). (C) Comparison with control protein classes: TF (ρ = 0.845, saturated at 72), Kinase (ρ = 0.429, ns), GPCR (ρ = −0.116, ns). (D) 3′UTR median length co-evolution with RBP diversity (ρ = 0.943, *p* = 0.0048). ∗ *p* < 0.05, ∗∗ *p* < 0.01, and ∗∗∗ *p* < 0.001; ns: not significant (Spearman’s rank correlation).

To investigate the relationship between RBP diversity and organismal complexity, we analyzed RBP family composition across six model organisms (Tables S1 and S2). Using a unified Pfam domain-based classification anchored to the EuRBPDB^20^ reference list of 686 RNA-binding domain families (see STAR Methods), RBP Pfam domain family diversity increased progressively from invertebrates to vertebrates: 397 families in *C. elegans*, 419 in *D. melanogaster*, 455 in *D. rerio*, 446 in *X. tropicalis*, 472 in *M. musculus*, and 469 in *H. sapiens* (Figure 1A). This diversity correlated strongly with neuronal count (Spearman’s ρ = 0.886, *p* = 0.019, *n* = 6; Figure 1B) and with genome size and cell type diversity (Figure 1C; Table S2).

To assess robustness, we performed bootstrap resampling (10,000 iterations; 95% CI [0.200, 1.000]; median ρ = 0.882; 0.3% of bootstrap samples yielded negative correlations) and leave-one-out validation (ρ = 0.800–0.900 regardless of which species was excluded; Figure S1). Effect size analysis confirmed that the difference between RBP and control protein correlations was large: Cohen’s d = 1.157 (RBP vs. kinase) and 1.861 (RBP vs. GPCR; Table S3). Pfam clan-level analysis (274–325 clans) yielded the identical correlation (ρ = 0.886, *p* = 0.019), confirming robustness across classification granularity (Figure S2).

To address phylogenetic non-independence, we performed phylogenetic generalized least squares (PGLS) analysis. The optimal Pagel’s λ was 0.0, and the correlation remained significant across λ values from 0 to 0.9 (Figure S3), confirming robustness to phylogenetic correction across a wide range of phylogenetic signal assumptions (Table S4).

To assess generalizability across a broader phylogenetic range, we extended the analysis to 13 metazoan species (the six primary species plus turtle, chicken, sea squirt, lancelet, honeybee, octopus, and mosquito) using a unified UniProt keyword-based RBP list (see STAR Methods; Table S5). Despite the lower resolution of this dataset, RBP family diversity remained significantly correlated with neuronal count (ρ = 0.554, *p* = 0.050, *n* = 13), supporting the generalizability of the finding beyond the six primary model organisms.

To examine whether 3′UTR regulatory capacity co-evolved with RBP diversity, we analyzed transcript region lengths using Ensembl BioMart annotations. Median 3′UTR length increased ∼8.9-fold from worm (163 nt) to human (1,444 nt) for RBPs and showed strong correlation with neural complexity (ρ = 0.943, *p* = 0.0048) in both RBPs and the full proteome (Figure 1D). In contrast, neither 5′UTR length (proteome: ρ = 0.371, ns; RBP: ρ = −0.029, ns) nor CDS length (proteome: ρ = 0.200, ns; RBP: ρ = 0.429, ns) showed significant correlations, confirming that 3′UTR expansion is specifically associated with neural complexity—consistent with previous observations (Table S6).^21^^,^^22^

### The RBP-complexity correlation is specific to RBPs

**Figure 2:**
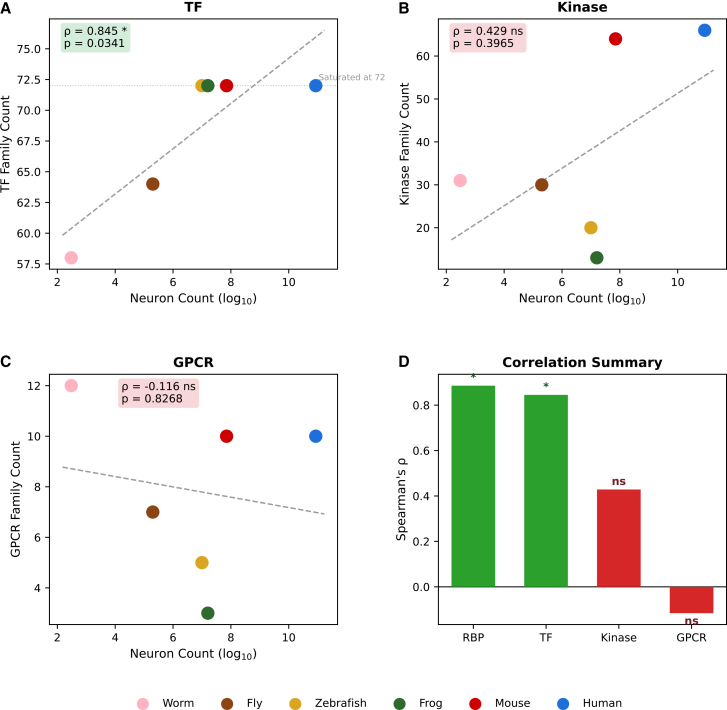
RBP-specific correlation with neural complexity (A) Transcription factor family count versus neuronal count (Spearman’s ρ = 0.845, *p* = 0.034, *n* = 6). TF family diversity saturates at 72 Pfam families across all four vertebrate species, indicating a ceiling effect rather than continuous diversification. (B) Protein kinase family count versus neuronal count (ρ = 0.429, ns). (C) GPCR family count versus neuronal count (ρ = −0.116, ns). Note the elevated GPCR family count in C. elegans, reflecting invertebrate-specific chemoreceptor expansion. (D) Summary bar plot of Spearman’s ρ values for RBP, TF, Kinase, and GPCR family diversity versus neuronal count. Green: significant (*p* < 0.05); red: not significant. Only RBP shows a statistically significant, continuous correlation with neural complexity. All correlations were computed using Pfam domain-based family classification. ∗ *p* < 0.05, ∗∗ *p* < 0.01, and ∗∗∗ *p* < 0.001; ns: not significant (Spearman’s rank correlation).

To assess specificity, we compared RBP patterns to three control protein classes using the same Pfam domain-based methodology (Table S7). Transcription factors showed a positive trend (ρ = 0.845, *p* = 0.034; Figure 2A), but this reflected complete saturation at 72 Pfam families across all four vertebrate species (zebrafish, frog, mouse, human, all = 72), rather than continuous diversification. In contrast, RBP Pfam families continued increasing within vertebrates (zebrafish: 455 → human: 469), indicating ongoing specialization. Kinases (ρ = 0.429, ns; Figure 2B) and GPCRs (ρ = −0.116, ns; Figure 2C) showed no significant correlation (Figure 2D).

This pattern—continuous RBP expansion versus TF saturation—suggests that RBP diversification reflects a qualitatively distinct evolutionary process, likely driven by increasing demands for post-transcriptional regulation rather than by genome-wide expansion shared with other regulatory proteins.

### Vertebrate RBP diversification is driven by domain-level expansion beyond canonical neural RBPs

**Figure 3:**
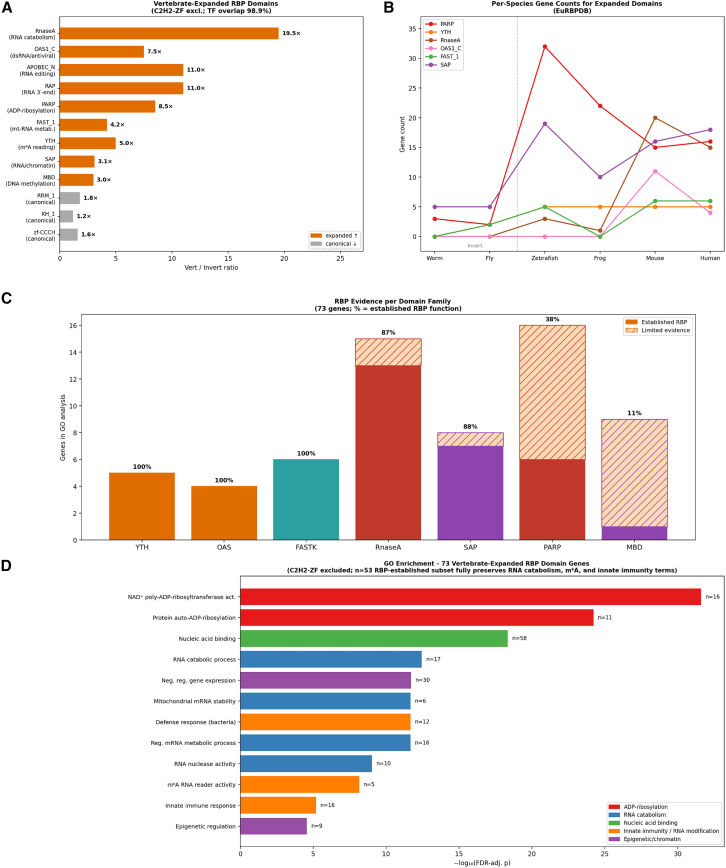
Domain-level expansion of RBP Pfam families in vertebrates (A) Vertebrate/invertebrate gene count ratios for nine RBP Pfam domain families showing ≥3-fold enrichment or vertebrate-specific emergence (C2H2-ZF excluded; 98.9% overlap with TFs). RNaseA (19.5×), APOBEC_N (11.0×), and RAP (11.0×) show the highest ratios; OAS1_C (7.5×), PARP (8.5×), YTH (5.0×), and SAP (3.1×) also show substantial enrichment. Vertebrate mean/invertebrate mean; pseudo-count 0.5 applied when invertebrate mean = 0. Canonical neural RBP domains (RRM_1, KH_1, zf-CCCH) show modest 1.2- to 1.8-fold expansion (shown for reference). (B) Per-species gene counts for six representative domains across six species (*C. elegans, D. melanogaster, D. rerio, X. tropicalis, M. musculus, H. sapiens*). Domain expansion occurred predominantly at the invertebrate-to-vertebrate transition; notably, the PARP domain expanded from a mean of 2.5 genes in invertebrates to 32 in zebrafish, while the YTH domain emerged from near-absence (0–1 gene) to consistent vertebrate representation (5 genes per species). The RNase A domain shows a mammalian-specific pattern: absent in invertebrates, rare in early vertebrates (frog: 1 gene, zebrafish: 3 genes), but expanded in mouse (20 genes) and human (15 genes). (C) RBP evidence classification for the seven domain families included in the GO analysis. Each bar shows total genes entering the GO query; shading indicates proportion with established RNA-binding evidence (solid) versus limited evidence (hatched). Domain families with 100% established evidence (YTH, OAS, FASTK) reflect high-confidence RBP activity, while PARP (40%) and MBD (14%) include non-RBP proteins. (D) Gene Ontology enrichment analysis of 73 vertebrate-expanded RBP domain genes (C2H2-ZF excluded). Top enriched terms: ADP-ribosylation (protein auto-ADP-ribosylation, FDR-corrected *p* = 6.0 × 10^−25^), RNA catabolism (RNA catabolic process, *p* = 3.6 × 10^−13^), and innate immune response (*p* = 6.2 × 10^−6^). Bar color indicates functional category (legend); n = number of query genes annotated to each term. Restricting to 53 genes with established RBP function (excluding 20 limited-evidence genes; C; Table S9) fully preserved RNA catabolism, m^6^A, and innate immunity enrichments (Δn = 0). See also Tables S6 and S7.

To identify which RBP Pfam domains drove the observed diversification, we calculated vertebrate/invertebrate expansion ratios for all canonical RBP domains using a unified Pfam classification across six species (Table S8). This domain-level analysis revealed nine functionally distinct Pfam domains showing ≥ 3-fold vertebrate enrichment or vertebrate-specific emergence (Figure 3A).

Notably, the most dramatically expanded domains were not canonical neural RNA-binding domains, but rather those involved in ADP-ribosylation (PARP domain, 8.5-fold), m^6^A RNA modification (YTH domain, 5.0-fold), and RNA 3′-end processing (RAP domain, 11.0-fold). Two domain families emerged as mammalian-specific: the RNase A domain (present in RNASE1–13 and related antimicrobial ribonucleases) and the OAS domain (oligoadenylate synthase, central to antiviral innate immunity). In contrast, domains harboring well-characterized neural RBPs—RRM_1 (containing CELF and ELAVL/Hu proteins), KH_1 (containing FMRP/FXR1), and zf-CCCH (containing MBNL proteins)—showed more modest expansion of 1.6- to 2.0-fold across the invertebrate-to-vertebrate transition.

Phylogenetic trajectory analysis confirmed that domain expansion occurred primarily at the invertebrate-to-vertebrate transition (Figure 3B). The PARP domain expanded from a mean of 2.5 genes in invertebrates to 32 in zebrafish, while the YTH domain emerged from near-absence in invertebrates (0–1 genes) to consistent vertebrate representation (5 genes per species). The RNase A domain showed a mammalian-specific pattern: absent in invertebrates, rare in early vertebrates (frog: 1 gene, zebrafish: 3 genes), but substantially expanded in mouse (20 genes) and human (15 genes).

To assess the functional relevance of vertebrate-expanded RBP domains, we classified each domain family by strength of RNA-binding evidence (Figure 3C and Table S9): Domains such as YTH, OAS, and FASTK show 100% established RBP function, while PARP (40%) and MBD (14%) include proteins with limited RNA-binding evidence. Gene Ontology enrichment analysis of all 73 vertebrate-expanded RBP domain genes (excluding C2H2 zinc finger proteins, which show 98.9% overlap with transcription factors; see Table S9) revealed significant enrichment for ADP-ribosylation (protein auto-ADP-ribosylation, adjusted *p* = 6.0 × 10^−25^), RNA catabolism (RNA catabolic process, adjusted *p* = 3.6 × 10^−13^), and innate immune response (adjusted *p* = 6.2 × 10^−6^, driven by OAS1/2/3 and RNASE family expansion; Figure 3D and Table S7). Restricting the analysis to the 53 genes with established RBP function (excluding the 20 limited-evidence genes shown in Figure 3C) fully preserved the RNA catabolism, m^6^A, and innate immunity terms (Δn = 0), confirming these reflect genuine post-transcriptional regulation. These findings reveal an unexpected picture of RBP diversification: Rather than being driven primarily by the expansion of neural RNA regulators, vertebrate RBP domain diversity reflects a broad functional expansion encompassing immunity, epigenetic regulation, and RNA modification. We propose that this multifunctional expansion of post-transcriptional regulatory capacity—across diverse biological processes—constitutes the molecular basis for the strong correlation between RBP family diversity and organismal complexity observed in Figure 1.

**Figure 4:**
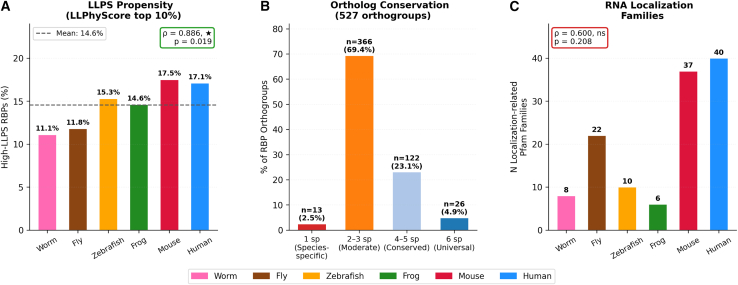
LLPS propensity, ortholog conservation, and spatial mRNA regulation (A) LLPS propensity across six species (LLPhyScore within-proteome top 10% threshold). High-LLPS RBP proportion shows a positive trend with neural complexity (range: 11.1–17.5%; ρ = 0.886, *p* = 0.019), while mean LLPS percentile rank is conserved (ρ = 0.600, ns), indicating that overall LLPS propensity is a fundamental conserved property with modest enrichment of high-LLPS RBPs in more complex organisms. (B) Ortholog conservation patterns across six species. Distribution of 527 canonical RBP orthogroups: species-specific (1 sp; *n* = 13, 2.5%), moderately conserved (2–3 sp; *n* = 366, 69.4%), broadly conserved (4–5 sp; *n* = 122, 23.1%), and universally conserved (6 sp; *n* = 26, 4.9%). (C) Number of Pfam domain families with significant GO enrichment for RNA localization (FDR <0.05) per species (range: 6–40). Positive trend with neural complexity not statistically significant (ρ = 0.600, *p* = 0.208). See also Figure 5 for IDR evolution analysis.

To test whether phase separation capacity was associated with domain expansion, we analyzed the relationship between domain size and LLPS propensity across all canonical domains. Expanded domains showed markedly diverse LLPS profiles: The YTH domain exhibited 100% high-LLPS membership, while the SAP domain showed 60%, and the RNase A domain showed only 3%. This diversity demonstrates that evolutionary domain expansion and LLPS propensity are independent properties, consistent with the conservation of overall LLPS capacity across species (Figure 4A).

To assess the conservation of RBP orthologs across the six species, we performed orthogroup analysis using OrthoFinder. Of 527 orthogroups containing canonical RBPs, 380 (72.1%) showed strict 1:1:1 single-copy conservation within their represented species, while 96 (18.2%) showed small expansions (≤2 copies; Table S10). Only 8 orthogroups (1.5%) showed large expansions (>5 copies), consistent with the domain-level expansion analysis above (Figure 4B). Additionally, GO enrichment analysis identified RBP Pfam families with RNA localization functions per species (Figure 4C and Table S11).

### LLPS propensity is evolutionarily conserved

Given the prominence of LLPS in RBP function and disease, we investigated whether LLPS propensity changed during evolution. Using LLPhyScore^23^ computed from full-length protein sequences for all RBPs across six species (13,071 RBP genes), we assessed within-proteome LLPS percentile ranks to enable cross-species comparison. The mean LLPS percentile rank was conserved across species (ρ = 0.600, *p* = 0.208, ns; Table S12), indicating that overall LLPS propensity does not scale with neural complexity. The proportion of RBPs in the top 10% of their respective proteomes showed a positive trend with neural complexity (range: 11.1–17.5%; ρ = 0.886, *p* = 0.019; Figure 4A), as did the top-20% threshold (ρ = 0.870, *p* = 0.024). This pattern reflects that while the overall LLPS level of the RBP repertoire is conserved, complex organisms harbor a modestly larger fraction of high-LLPS RBPs.

For cross-method validation, we analyzed pre-computed PhaSePred predictions^24^ for five species (*X. tropicalis* excluded due to data unavailability). Coverage varied substantially by species (human: 99%, mouse: 91%, worm: 55%, fly: 42%, zebrafish: 19%), reflecting PhaSePred’s restriction to SwissProt-reviewed proteins. catGRANULE^25^ (ρ = 0.700, ns) and PLAAC^26^ (ρ = 0.400, ns) showed no significant correlations. PScore^27^ showed a nominal correlation (ρ = 0.900, *p* = 0.037), but this should be interpreted cautiously given the substantial coverage bias (zebrafish 19% vs. human 99%) (Figure S4).

These findings indicate that the mean LLPS level of the RBP repertoire is evolutionarily conserved, while a modestly larger fraction of high-LLPS RBPs accumulates in more complex organisms. This is consistent with LLPS representing a fundamental property of many RBPs rather than a mechanism specifically recruited during vertebrate diversification, with the ancestral RBP toolkit already harboring substantial phase-separation capacity.

### Intrinsically disordered regions expand with neural complexity

**Figure 5:**
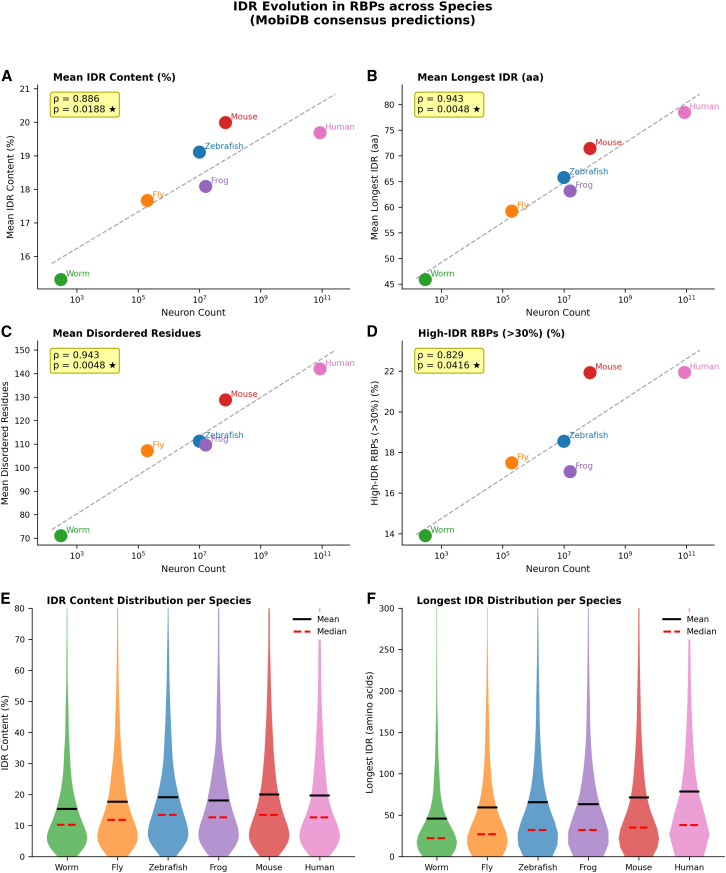
Intrinsically disordered region (IDR) evolution in RBPs across species (A–D) Mean IDR content (%), mean longest IDR length (aa), mean total disordered residues (aa), and proportion of high-IDR RBPs (>30% disordered) across six species plotted against neuronal count (log_10_ scale). Data from MobiDB consensus predictions (*n* = 12,222 RBPs). Spearman’s correlations: IDR content ρ = 0.886 (*p* = 0.019); longest IDR ρ = 0.943 (*p* = 0.005); disordered residues ρ = 0.943 (*p* = 0.005); high-IDR RBPs ρ = 0.812 (*p* = 0.050). (E–F) Violin plots show the within-species distribution of IDR content (%) and longest IDR length (aa). Broad distributional overlap across species is consistent with a conserved IDR architecture at the population level, while mean values increase progressively with neural complexity.

To investigate whether RBP structural properties evolved with organismal complexity, we analyzed intrinsically disordered regions (IDRs) using MobiDB consensus predictions across 12,222 RBPs from all six species. The proportion of disordered residues showed a significant increase across species (mean IDR content 15.3–20.0%, ρ = 0.886, *p* = 0.019; Figure 5A), the absolute length of the longest IDR per protein increased significantly with neural complexity: mean 45.9 aa in *C. elegans* to 78.5 aa in *H. sapiens* (ρ = 0.943, *p* = 0.005; Figure 5B). Similarly, the total number of disordered residues per protein increased from 71.1 to 142.0 aa (ρ = 0.943, *p* = 0.005; Figure 5C and Table S13.

Both the proportion and absolute length of IDRs increased with neural complexity, with absolute IDR length showing a stronger correlation (ρ = 0.943) than IDR content percentage (ρ = 0.886) (Figures 5D–5F). This pattern suggests that longer proteins in more complex organisms harbor disordered stretches that are longer in absolute terms, potentially providing expanded interaction surfaces for protein-protein and protein-RNA interactions. Thus, increased regulatory capacity in complex organisms is achieved not only through modest increases in the proportion of disorder, but more prominently through the expansion of absolute disordered segments that can accommodate additional regulatory interactions. Notably, many RBPs implicated in neurological diseases—including TDP-43, FUS, and hnRNPA2B1^28^^,^^29^—harbor extended IDRs that drive pathological aggregation, suggesting that the evolutionary expansion of IDR length in vertebrate RBPs may represent both a functional innovation and a vulnerability to neurodegenerative disease.

### Non-canonical RBPs and RBP-transcription factor overlap

To assess whether our findings were limited to canonical RNA-binding domain-containing proteins, we analyzed non-canonical RBPs (experimentally confirmed RBPs lacking recognizable RBDs) using RBPWorld^30^ for five species (*X. tropicalis* was excluded due to data unavailability). Non-canonical RBP counts correlated positively with neural complexity (ρ = 0.900, *p* = 0.037), paralleling the canonical RBP trend. When canonical and non-canonical RBPs were analyzed together, the total RBP count also correlated significantly with neural complexity (ρ = 0.900, *p* = 0.037; Table S14), confirming that the relationship between RBP diversity and neural complexity is robust to the inclusion of non-canonical RBPs.

We also assessed potential overlap between RBPs and transcription factors, as some proteins function in both capacities. RBP-TF overlap averaged 2.6–8.3% of canonical RBPs across species (excluding zebrafish, where zf-C2H2 multi-annotation inflated apparent overlap to 16.0%). In humans, 194 of 2,961 RBPs (6.6%) overlapped with TFs annotated in AnimalTFDB, of which 86 carried zf-C2H2 domains. These results confirm that the two protein classes are largely distinct, and that our RBP diversity measures are not confounded by TF annotation overlap (Table S15).

## Discussion

We present a systematic comparative analysis of RBP family diversity across six metazoan species, demonstrating that RBP Pfam domain family diversity correlates strongly and specifically with neural complexity (ρ = 0.886, *p* = 0.019). This correlation is robust to bootstrap resampling, leave-one-out validation, and phylogenetic correction, and is specific to RBPs—transcription factors show vertebrate saturation rather than continuous diversification, while kinases and GPCRs show no significant correlation.

Our finding that transcription factor family diversity saturates at 72 Pfam families across all four vertebrate species, while RBP families continue increasing (455 in zebrafish to 469 in humans), suggests a qualitative difference in evolutionary strategy. This distinction aligns with the view that post-transcriptional regulation provides an additional regulatory layer in vertebrates beyond what transcriptional control alone can achieve—particularly in neurons, which require spatially restricted, rapid, and activity-dependent gene regulation.^5^^,^^6^^,^^7^

Our findings regarding LLPS propensity reveal a nuanced picture. The mean LLPS percentile rank of the RBP repertoire is conserved across species (ρ = 0.600, ns), indicating that LLPS is a fundamental, ancestral property of RBPs. At the same time, the proportion of high-LLPS RBPs (top 10%) shows a modest positive trend with neural complexity (ρ = 0.886, *p* = 0.019), suggesting that more complex organisms have selectively expanded their high-LLPS RBP fraction. This pattern—conserved mean propensity with enrichment at the high end—is consistent with LLPS representing a core feature of the ancestral RBP toolkit, with subsequent vertebrate expansion proportionally increasing both LLPS-capable and LLPS-independent RBPs while modestly favoring phase-separation-competent proteins.

In contrast, both the absolute length and overall proportion of IDRs increase with neural complexity, with absolute IDR length showing the stronger correlation (ρ = 0.943 vs. ρ = 0.886 for IDR content percentage). This pattern suggests that longer proteins in complex organisms harbor disordered stretches that are longer in absolute terms, potentially enabling more complex regulatory assemblies. The combination of conserved LLPS propensity and expanding absolute IDR length may represent complementary mechanisms: conserved phase-separation capacity for forming RNA-protein condensates, paired with increasingly elaborate disordered interaction interfaces.

The co-evolution of 3′UTR length and RBP family diversity—both correlating strongly with neural complexity (3′UTR length: ρ = 0.943; RBP family diversity: ρ = 0.886) while 5′UTR and CDS lengths do not—supports a model of coordinated regulatory co-evolution. Longer 3′UTRs provide increased binding sites for the expanding RBP repertoire, consistent with previous proposals of co-evolutionary optimization of post-transcriptional regulatory capacity.^21^^,^^22^^,^^31^^,^^32^^,^^33^^,^^34^

Our domain-level analysis challenges the prevailing view that RBP diversification in vertebrates is primarily driven by the expansion of neural RNA regulators. While canonical neural RBP domains (RRM_1, KH_1, zf-CCCH) do show modest vertebrate enrichment of 1.2- to 1.8-fold, the most dramatically expanded domains serve functions in ADP-ribosylation and DNA repair (PARP) and innate immunity (OAS), epitranscriptomic regulation (YTH/m^6^A reading), and RNA catabolism—not synaptic plasticity per se. This observation suggests a reinterpretation: Rather than neural complexity driving the expansion of neural RBPs, the correlation between RBP diversity and neural complexity (ρ = 0.886, *p* = 0.019) may reflect a broader co-evolutionary relationship between organismal complexity and post-transcriptional regulatory capacity across multiple biological systems. PARP domain proteins exemplify this complexity: While most PARP family members (PARP3–8, TNKS1/2) primarily function in DNA repair and telomere maintenance, PARP1 and PARP12/14 have established roles in RNA metabolism and neuronal activity.^35^ YTH domain proteins mediate m^6^A-dependent mRNA fate decisions—including translational activation and mRNA stability regulation—that have been linked to neural development and synaptic function. OAS proteins participate in innate immune responses that intersect with neuroinflammation. Notably, the strong GO enrichment for innate immunity and defense response terms observed in our analysis may at least in part be attributable to the RNase A family (RNASE1–13, ANG), whose mammalian-specific expansion is likely a driver of these terms and reflects lineage-specific diversification into antimicrobial and host-defense functions rather than neural functions per se. Thus, the domains most dramatically expanded in vertebrates may contribute to neural complexity indirectly—through cellular stress response, epitranscriptomic regulation, genome maintenance, and, in the case of RNase A family proteins, antimicrobial defense—rather than through direct synaptic RNA regulation. This perspective aligns with the broader finding that RBP expansion is not simply a scaled-up version of invertebrate RBP repertoires, but a qualitatively different regulatory landscape that may underlie the multifaceted complexity of vertebrate biology.

The correlation between non-canonical RBP counts and neural complexity (ρ = 0.900, *p* = 0.037) provides independent support for our main finding, which is not dependent on domain-based classification. Non-canonical RBPs—experimentally confirmed RBPs that lack recognizable RNA-binding domains—likely reflect the acquisition of distinct RNA-interaction interfaces through intrinsic disorder or surface electrostatics rather than canonical domain recruitment. Their parallel expansion with neural complexity suggests that vertebrate nervous systems have diversified their post-transcriptional regulatory capacity through multiple evolutionary routes, with domain-family diversification and non-canonical RBP acquisition representing complementary, rather than competing, mechanisms. This interpretation is consistent with the broader view that the complexity of neuronal RNA regulation cannot be attributed to any single molecular innovation, but emerges from the concerted expansion of multiple RNA-protein interaction strategies.

In conclusion, we establish RBP Pfam domain family diversity as a specific, robust molecular correlate of neural complexity, achieved through vertebrate-specific acquisition of additional RBP families rather than the expansion of the ancestral toolkit, and accompanied by increasing absolute disordered region length and a modest enrichment of high-LLPS RBPs in more complex organisms.

### Limitations of the study

Our family diversity metric counts distinct Pfam domain families but does not capture paralog-level expansion within families. Neuronal counts are imprecise estimates for non-mammalian species. The correlation with genome size (ρ = 0.943) suggests that some of the RBP expansion may reflect general genomic complexity rather than neural-specific selection. Future work examining species with divergent brain size-genome size relationships will be needed to dissociate these effects. The non-canonical RBP and IDR analyses are limited to five species, as *X. tropicalis* data are not available in RBPWorld or MobiDB at sufficient coverage; inclusion of additional amphibian data in future work would strengthen the generalizability of these findings.

## Resource availability

### Lead contact

Further information and requests for resources should be directed to the lead contact, Kyota Yasuda.

### Materials availability

This study did not generate new biological materials.

### Data and code availability

#### Data

All data reported in this paper are derived from publicly available databases (EuRBPDB, RBPWorld, AnimalTFDB 4.0, MobiDB, Ensembl BioMart, UniProt, PhaSePred, LLPhyScore). Processed datasets supporting this study are publicly available at Zenodo (DOI: https://doi.org/10.5281/zenodo.18002633). Data reported in this paper will be shared by the lead contact upon request.

#### Code

All analysis scripts used in this study are publicly available at GitHub (https://github.com/Kyotay-12/rbp-evolution-analysis) and archived at Zenodo (DOI: https://doi.org/10.5281/zenodo.18002633).

#### Other items

Any additional information required to reanalyze the data reported in this paper is available from the lead contact upon request.

## Acknowledgments

This work was supported by JSPS KAKENHI Grant-in-Aid for Scientific Research (C) (grant no. 23K05147). The author thanks the EuRBPDB, AnimalTFDB, MobiDB, and RBPWorld database teams for providing publicly accessible data resources.

## Author contributions

K.Y. conceived the study, performed all analyses, and wrote the manuscript.

## Declaration of interests

The author declares no competing interests.

## Declaration of generative AI and AI-assisted technologies in the writing process

During the preparation of this work, the author used Claude (Anthropic) in order to assist with statistical analysis verification, figure preparation, literature organization, and manuscript editing. After using this tool, the author reviewed and edited the content as needed and takes full responsibility for the content of the published article.

## STAR★Methods

### Key resources table

**Table:** 

REAGENT or RESOURCE	SOURCE	IDENTIFIER
**Deposited data**		

RBP family classifications and complexity metrics (this paper)	This paper	GitHub: https://github.com/Kyotay-12/rbp-evolution-analysis
Archived dataset	This paper	Zenodo: DOI: https://doi.org/10.5281/zenodo.18002633

**Databases and online resources**		

EuRBPDB	Liao et al.^20^	http://EuRBPDB.syshospital.org
RBPWorld	Liao et al.^30^	http://research.gzsys.org.cn/eurbpdb2/
AnimalTFDB 4.0	Shen et al.^36^	http://bioinfo.life.hust.edu.cn/AnimalTFDB4/
MobiDB	Piovesan et al.^37^	https://mobidb.bio.unipd.it/
Ensembl BioMart (release 115)	Ensembl	https://www.ensembl.org/biomart
LLPhyScore	Cai et al.^23^	GitHub: fukunagatsu/LLPhyScore
PhaSePred	Wang et al.^24^	https://phasepred.net
UniProt REST API	UniProt Consortium	https://www.uniprot.org/help/api
OrthoFinder v2.5	Emms and Kelly^38^	https://github.com/davidemms/OrthoFinder
TimeTree	Kumar et al.^39^	https://timetree.org

**Software and algorithms**		

Python 3.9	Python Software Foundation	https://www.python.org/; RRID:SCR_008394
SciPy	Virtanen et al., 2020^40^	https://scipy.org/; RRID:SCR_008058
NumPy	Harris et al., 2020^41^	https://numpy.org/; RRID:SCR_008633
Pandas	McKinney, 2010^42^	https://pandas.pydata.org/; RRID:SCR_018214
Matplotlib	Hunter, 2007^43^	https://matplotlib.org/; RRID:SCR_008624
Seaborn	Waskom, 2021^44^	https://seaborn.pydata.org/; RRID:SCR_018132
Custom analysis scripts	This paper	GitHub: https://github.com/Kyotay-12/rbp-evolution-analysis

### Experimental model and study participant details

This study is entirely computational and did not involve experimental models or study participants. No animals, cell lines, human subjects, or microorganisms were used. All analyses were performed using publicly available databases and previously published datasets. Sex and gender were not applicable to this study.

### Method details

#### RBP identification

RBPs for the six primary species were identified from EuRBPDB,^20^ a comprehensive, manually curated database providing RBP annotations based on RNA-binding domain (RBD) analysis across 162 eukaryotic species. The EuRBPDB files (∗_RBP.txt) were used for all primary analyses: *C. elegans* (1,442 genes), *D. melanogaster* (1,633), *D. rerio* (2,349), *X. tropicalis* (1,758), *M. musculus* (2,995), *H. sapiens* (2,961). These six species were selected because they fulfill three criteria simultaneously: (1) coverage by EuRBPDB with complete RBP gene lists, (2) coverage by AnimalTFDB 4.0 with transcription factor family annotations, and (3) sufficient UniProt reviewed entries to support Kinase and GPCR control protein analyses—enabling a fully unified four-protein-type comparison across the same set of species (Table S2).

Neuronal counts were used as a proxy measure of neural complexity. Values were obtained from published estimates: *C. elegans* (302 neurons),^34^ *D. melanogaster* (200,000),^45^ *D. rerio* (10,000,000),^46^ *X. tropicalis* (16,000,000),^47^ *M. musculus* (71,000,000),^48^ *H. sapiens* (86,000,000,000).^49^^,^^50^

For the expanded 13-species analysis, RBPs were identified using the UniProt keyword search (keyword:KW-0694, RNA-binding)^51^ for all 13 species, including the six primary species and seven additional taxa: Pelodiscus sinensis (turtle), Gallus gallus (chicken), Ciona intestinalis (sea squirt), Branchiostoma lanceolatum (lancelet), Apis mellifera (honeybee), Octopus bimaculoides (octopus), and Aedes aegypti (mosquito). To ensure cross-species comparability, all 13 species were processed with the same UniProt keyword pipeline rather than mixing EuRBPDB and UniProt sources. Results from this expanded analysis are reported in Table S5.

#### RBP family classification

Family classification was performed using a unified Pfam domain-based approach. Gene symbols from EuRBPDB were mapped to UniProt accessions via the UniProt REST API (gene_exact search). Pfam domain annotations were retrieved for each protein.^52^ Proteins were assigned to families by matching Pfam domains against the EuRBPDB reference list of 686 RNA-binding domain families. When multiple Pfam domains matched different families, the family with the highest gene count in EuRBPDB was prioritized. Proteins with no matching Pfam domain were classified as non-canonical. For the Human RBP dataset (which lacks pre-assigned family information in EuRBPDB), family assignments were inferred from Pfam domain–family mappings derived from the five non-human species. Robustness was confirmed by Pfam clan-level analysis (CL0001–CL9999 hierarchical groupings from InterPro), which yielded an identical correlation (ρ = 0.886, *p* = 0.019).

#### Control protein classification

Transcription factor families were assigned using tf_family annotations from AnimalTFDB 4.0.^36^ Kinase and GPCR families were assigned by Pfam domain using UniProt reviewed proteins with keyword annotations KW-0418 (Kinase)^53^ and KW-0297 (G-protein coupled receptor),^54^ respectively. Pfam domain assignments were based on the Pfam database.

#### IDR analysis

Intrinsically disordered regions were predicted using the MobiDB database,^37^ which provides consensus disorder predictions integrating multiple algorithms. For each RBP, disorder annotations were retrieved via the MobiDB REST API, extracting the percentage of disordered residues, the length of the longest contiguous IDR, and the total number of disordered residues. Species-level statistics were computed as means across all RBPs with available predictions (12,222 of 13,138 RBPs, 93.0% coverage).

#### LLPS analysis

LLPS propensity was assessed using LLPhyScore,^23^ computed from full-length protein sequences obtained from UniProt for all RBPs (13,071 genes after gene-level aggregation using maximum scores across isoforms). Because LLPhyScore was trained on the human proteome, raw scores are not directly comparable across species. We therefore computed within-proteome percentile ranks for each species independently. The proportion of RBPs in the top 10% and 20% of their respective proteomes was used as the primary metric. For cross-validation, pre-computed PhaSePred^24^ predictions were obtained for five species (*X. tropicalis* excluded due to data unavailability). PhaSePred coverage was: human 99.2%, mouse 90.9%, worm 54.5%, fly 42.3%, zebrafish 18.8%, reflecting restriction to SwissProt-reviewed proteins.

#### 3′UTR/CDS/5′UTR length analysis

Transcript region lengths were obtained from Ensembl BioMart (release 115)^55^ for all protein-coding genes in the six primary species. For each gene, the 5′UTR length, CDS length, 3′UTR length, and total mRNA length were extracted. When multiple transcripts existed per gene, the transcript with the longest 3′UTR was retained for RBP-specific analyses. Proteome-wide medians were computed for comparison. RBP-specific analyses filtered for genes present in the EuRBPDB database.

#### Non-canonical RBP analysis

Non-canonical RBPs were identified using RBPWorld for five species (*X. tropicalis* excluded due to data unavailability). Canonical RBPs were defined as those containing recognized RNA-binding domains; non-canonical RBPs were experimentally identified RNA-binding proteins lacking such domains. RBP–TF overlap was assessed by intersecting EuRBPDB gene symbols with AnimalTFDB gene symbols for each species.

#### Orthogroup analysis

Orthologous gene groups were identified using OrthoFinder v2.5 with default parameters (DIAMOND alignment, MCL clustering).^38^ Input sequences were canonical RBP protein sequences (FASTA format) retrieved from UniProt for all six primary species. Orthogroups were classified by conservation level: species-specific (present in 1 species), moderate (2–3 species), conserved (4–5 species), and universal (all 6 species).

### Quantification and statistical analysis

All correlations were calculated using Spearman’s rank correlation coefficient (scipy.stats.spearmanr). Bootstrap resampling was performed with 10,000 iterations sampling 6 species with replacement. Leave-one-out validation computed correlations with each species removed in turn. Cohen’s d was computed using Fisher z-transformation of Spearman correlations: d = |z(ρ_RBP) − z(ρ_control)|/SE, where SE = √(2/(n−3)) and *n* = 6. Phylogenetic Generalized Least Squares (PGLS) regression was implemented following Martins & Hansen (1997)^56^ using custom Python scripts (scipy, numpy) with a time-calibrated phylogenetic tree from TimeTree.^39^ Pagel’s λ was optimized by maximum likelihood. All analyses used Python 3.10 with pandas, scipy, numpy, and matplotlib.

## Supplemental information

Document S1. Figures S1–S4

Table S1. Spearman’s rank correlations — RBP family diversity vs. neural complexity, related to Figure 1

Table S2. Species and dataset information, related to Figure 1

Table S3. Statistical robustness analyses, related to Figure 1

Table S4. Phylogenetic generalized least squares (PGLS) analysis, related to Figure S3

Table S5. Extended 13-species taxonomic validation, related to Figure S1

Table S6. Transcript region length correlations with neural complexity, related to Figure 1

Table S7. Family diversity by protein type and species, related to Figure 2

Table S8. Vertebrate-expanded RBP Pfam domains, related to Figure 3

Table S9. GO enrichment of vertebrate-expanded RBP domains, related to Figure 3

Table S10. RBP ortholog conservation, related to Figure 4

Table S11. RBP Pfam families with RNA localization GO enrichment, related to Figure 4

Table S12. LLPS propensity analysis, related to Figure 4

Table S13. Intrinsically disordered region (IDR) analysis, related to Figure 5

Table S14. Non-canonical RBP analysis, related to Figure 5

Table S15. RBP-transcription factor gene overlap, related to Figure 5
