# Supplementary material for: RNA-binding protein family diversification correlates with neural complexity across metazoan evolution
Source: iScience. 2026 Apr 17;29(5):115766. doi: 10.1016/j.isci.2026.115766 (PMC13157009; doi:10.1016/j.isci.2026.115766)

iScience, Volume *29*

Supplemental information

RNA-binding protein family diversification correlates with neural complexity across metazoan evolution

Kyota Yasuda

Supplementary Figure 1. Extended Taxonomic Validation and Statistical Robustness.

(A) RBP family diversity versus neuronal count across 13 metazoan species (Spearman ρ = 0.554, p = 0.050, n = 13), including sea squirt, lancelet, mosquito, honeybee, turtle, chicken, and octopus in addition to the six primary species. (B) Bootstrap resampling distribution (10,000 iterations): 95% CI [0.200, 1.000], median ρ = 0.882, 0.3% negative correlations. (C) Leave-one-out validation: ρ = 0.800–0.900 across all six subsets.

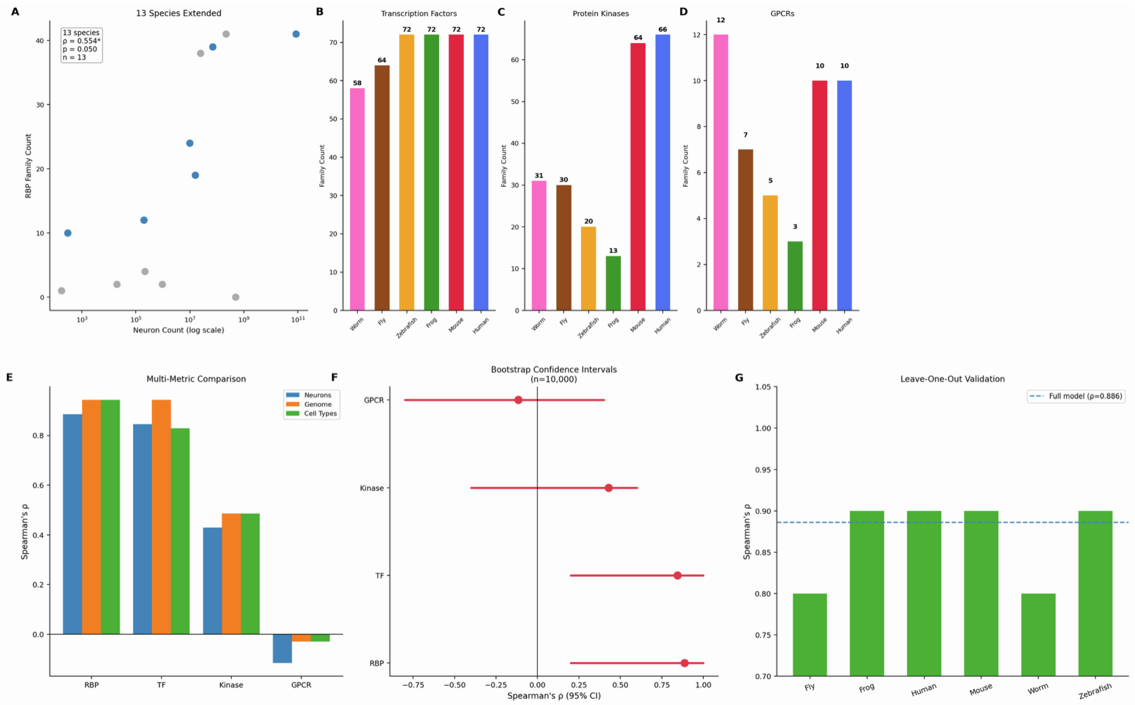

Supplementary Figure 2. Family-Level Correlation Distribution.

Distribution of Spearman ρ values for individual RBP Pfam domain family counts versus neuronal count (n = 6 species). Each point represents one Pfam domain family. The right-skewed distribution indicates that the diversity-complexity correlation reflects contributions from multiple families. Families represented in fewer than three species are excluded. Dashed line: ρ = 0.

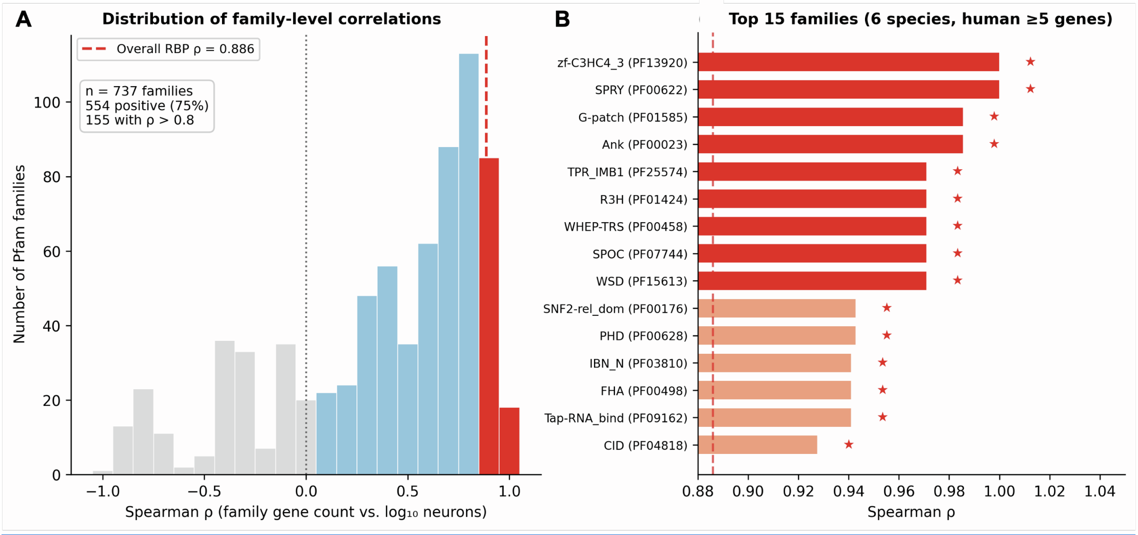

Supplementary Figure 3. Phylogenetic Generalized Least Squares (PGLS) Sensitivity Analysis.

PGLS regression of RBP family diversity versus neuronal count under varying phylogenetic signal assumptions (Pagel's λ). Optimal λ = 0.0 (maximum likelihood), indicating minimal phylogenetic signal. Under conservative Brownian motion (λ = 1), RBP retains a positive trend (p = 0.153). Branch lengths from TimeTree. See also Supplementary Table S4.

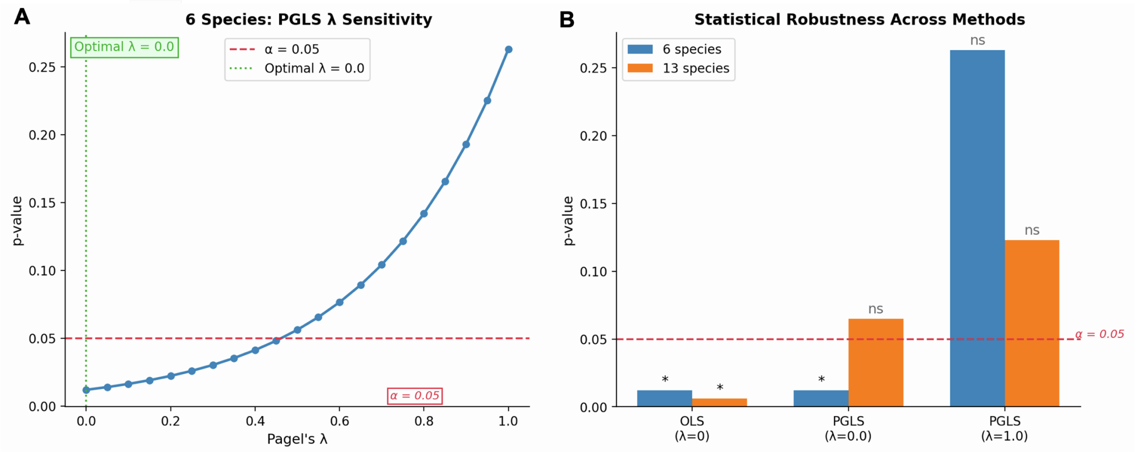

Supplementary Figure 4. LLPS Propensity — Multi-Predictor Validation.

Proportion of high-LLPS RBPs across species by four prediction methods: (A) catGRANULE (Bolognesi et al., 2016; ρ = 0.700, ns), (B) PScore (Vernon et al., 2018; ρ = 0.900, p = 0.037; note coverage bias: zebrafish 19% vs. human 99%), (C) PLAAC (Lancaster et al., 2014; ρ = 0.400, ns). PhaSePred predictions (Wang et al., 2022) for five species (X. tropicalis excluded). Mean LLPS percentile rank is conserved (LLPhyScore: ρ = 0.600, ns); high-LLPS RBP fraction (top 10%) shows positive trend (ρ = 0.886, p = 0.019). See also Supplementary Table S12.

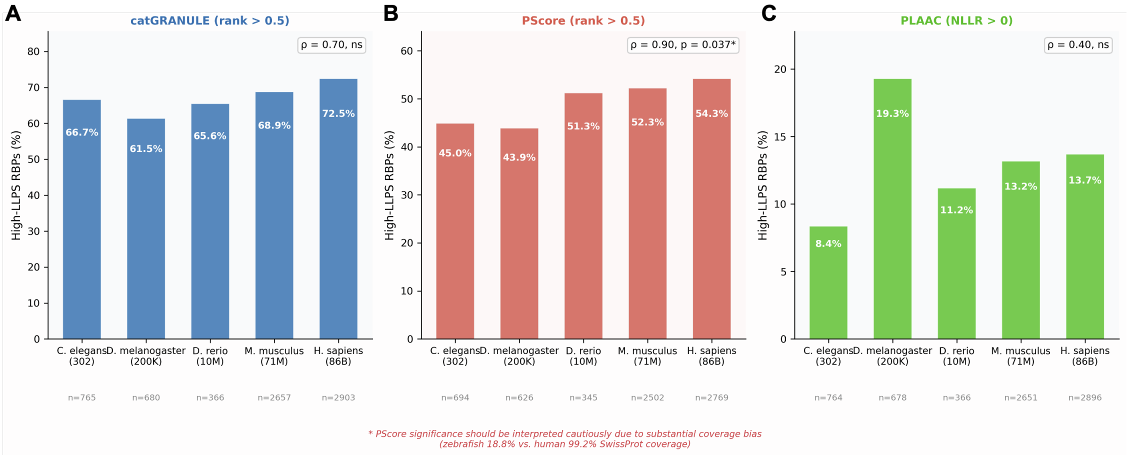

Supplement: Document S1. Figures S1–S4 [file mmc1.pdf]
